# Patient safety associated with the surgical treatment of bone and soft tissue tumours during the COVID-19 pandemic—results from an observational study at the Oxford Sarcoma Service

**DOI:** 10.1007/s00264-020-04736-1

**Published:** 2020-07-29

**Authors:** Raja Bhaskara Rajasekaran, Sanjeev Kotecha, Duncan Whitwell, Thomas D. A. Cosker, Paul Critchley, Charles Anton Fries, David Pigott, Christopher L. M. H. Gibbons, Andrew Carr

**Affiliations:** 1 The Oxford Bone and Soft tissue tumour service, Nuffield Department of Orthopaedics, Rheumatology and Musculoskeletal Sciences (NDORMS), Nuffield Orthopaedic Centre, Windmill Road, Oxford, OX37LD UK; 2 Nuffield Department of Orthopaedics, Rheumatology and Musculoskeletal Sciences, Botnar Research Centre, University of Oxford, Oxford, OX3 7LD UK

**Keywords:** COVID-19, Sarcoma, Bone tumour, Cancer surgery

## Abstract

**Purpose:**

Deferring cancer surgery can have profound adverse effects including patient mortality. During the COVID-19 pandemic, departmental reorganisation and adherence to evolving guidelines enabled provision of uninterrupted surgical care to patients with bone and soft tissue tumours (BST) in need of surgery. We reviewed the outcomes of surgeries on BST during the first two months of the pandemic at one of the tertiary BST centres in the UK.

**Materials and methods:**

Between 12 March 2020 and 12 May 2020, 56 patients of a median age of 57 years (18–87) underwent surgery across two sites: index hospital (*n* = 27) and COVID-free facility (*n* = 29). Twenty-five (44.6%) patients were above the age of 60 years and 20 (35.7%) patients were in ASA III and ASA IV category. The decision to offer surgery was made in adherence with the guidelines issued by the NHS, BOOS and BSG.

**Results:**

At a minimum follow-up of 30 days post-surgery, 54 (96.4%) patients were recovering well. Thirteen patients (23.2%) had post-operative complications which included four (7.1%) patients developing pulmonary embolism. The majority of complications (12/13 = 92.7%) occurred in ASA III and IV category patients. Four (7.1%) patients contracted COVID-19, of which three required escalation of care due to pulmonary complications and two (3.6%) died. Patients < 60 years of age had significantly less complications than those > 60 years (*p* < 0.001). Patients operated on in the COVID-free facility had fewer complications compared with those operated on at the index hospital (*p* < 0.027).

**Conclusion:**

In spite of the favourable results in majority of our patients, our study shows that patients with sarcoma operated at the height of the pandemic are at a risk of contracting COVID-19 and also having associated with mortality. The use of a COVID-free facility, surgery in patients < 60 60 years and in ASA I & II category are associated with better outcomes. If a second wave occurs, a serious consideration should be given to ways of minimising the risk of contracting COVID-19 in these vulnerable patients either by using COVID-free facilities or delaying treatment until peak of infection has passed.

## Introduction

The COVID-19 (SARS-CoV-2) pandemic has caused an unforeseen demand on healthcare systems across the globe. While elective surgery across all specialties have been postponed to prevent patients from contracting the disease and to avoid overwhelming healthcare facilities, this is not viable with cancer surgery [1–3]. Postponing cancer surgery can have adverse effects and can even compromise the lives of patients. Therefore, a prudent approach involving assessment of infrastructure and selection of appropriate cases for surgery is of utmost importance for cancer services to provide uninterrupted care. Early results from across the globe have shown that cancer patients, who are immunocompromised, are at an increased risk of serious complications related to COVID-19 compared with the rest of the population [4–6]. Numerous guidelines on how to provide effective cancer treatment have been issued during this pandemic providing guidance on restructuring services to provide unhindered treatment for patients [7–9]. However, data on outcomes of cancer surgeries performed during the pandemic is scant. Predictable and anticipated outcomes are required to plan ahead while managing all malignancies including rare cancers like pancreas and leukaemia, as well as bone and soft tissue tumours (BST), and would aid in formulating evidence-based guidelines to provide favourable results and more importantly minimise patient morbidity and mortality.

The Oxford Sarcoma Service, one of the five nationally approved centres in the UK for the management of both primary soft tissue and bone sarcomas, was quickly restructured and reorganised based on the evolving guidelines issued by the NHS to provide effective treatment to patients with BST during the pandemic [8, 10]. In the first two months, which included the peak of the pandemic in the UK, we performed surgery on 56 patients with BST. This article reports the outcomes of these cases and discusses the implications for BST surgery services during this pandemic.

## Materials and methods

Following the declaration of COVID-19 as a pandemic by the World Health Organization on 12 March 2020 [11], the decision to defer elective surgery was taken by the Oxford University Hospitals NHS Foundation Trust on 16 March 2020 in an attempt to avoid overwhelming the local healthcare system and prevent patients from contracting the disease in the hospital. The Nuffield Orthopaedic Centre (NOC), an index hospital for elective surgery and BST surgery in the Trust, was reorganised to manage fracture care in the elderly and rehabilitation of trauma patients following surgery, which involved cross-site transfer of patients. Due to redeployment of anaesthetists and healthcare personnel to manage COVID-19 patients requiring management in intensive care units, surgical theatre workflow pattern had to be restructured to provide continued service in cancer care. A non-public hospital in Oxford was effectively nationalised by the NHS at the start of the pandemic to manage COVID-negative cases, which needed surgery on an urgent basis with access to ITU. This was designated as a COVID-free facility. All patients requiring surgery and who had been on waiting lists were triaged as per guidelines issued by the NHS, BOOS and BSG to manage cancer patients requiring acute treatment [8, 9]. Patients were categorised as follows:

- Priority level 1a: *Emergency operation needed within 24 h to save life*
- Priority level 1b: *Urgent surgery needed within 72 h*
- Priority level 2: *Elective surgery to save life/prevent disease progression beyond operability*
- Priority level 3: *Elective surgery which can be delayed for 10–12 weeks and will have no predicted negative outcome*

Surgery that fell under priority level 1a, 1b and 2 and patients who were likely to benefit from a potentially curative cancer surgery were listed. Priority level 3 surgeries were postponed to a suitable time following the pandemic.

Asymptomatic patients, unless they had an international travel history or contact history, were not tested pre-operatively for COVID-19 until the first week of April 2020. From 8 April 2020 onwards, all pre-operative patients (*n* = 39) were subjected to nasopharyngeal and oropharyngeal swab test subjected to polymerase chain reaction (PCR) test for COVID-19, 72 h prior to surgery. Irrespective of the COVID-19 status of the patient, all surgery during the study period were performed using level 2 PPE with the involved healthcare professionals following strict protocols of ‘donning’ and ‘doffing’ prior to and after surgery. All patients received prophylaxis for DVT post-surgery as per NICE guidelines. All patients were followed up for a minimum period of 30 days post-surgery and their outcomes were analysed. Complications were assessed as per Clavien-Dindo system [11]. Pulmonary complications were defined as pneumonia, acute respiratory distress syndrome (ARDS) or unexpected postoperative ventilation as these were the most frequently described complications in patients affected with COVID-19 [12]. Simple descriptive statistics was used to analyse the results. Fisher’s exact tests were performed for statistical comparisons and results were considered significant at *p* < 0.05.

## Results

Between 12 March 2020 and 12 May 2020, 56 patients with a median age of 57 years (18–87) with BST were operated on by the Oxford Sarcoma Service. Twenty-seven (48.2%) patients were operated at the index hospital (NOC) and 29 (51.8%) patients were operated upon at the designated COVID-19 free facility.

Twenty-five (44.6%) patients were above 60 years and 31 (55.4%) patients under 60 years formed the study group. The majority of patients (*n* = 31) were in ASA II (55.3%), followed by 18 (32.1%) in ASA III, five (8.9%) in ASA I and two (3.6%) in ASA IV. Nineteen (33.9%) patients underwent an en bloc resection for bone tumours with 12 patients among them requiring prosthesis replacement and seven patients requiring reconstruction. Two (3.6%) patients underwent only en bloc resections and two (3.6%) metastatic fractures involving the hip joint was operated. Thirty-three (58.9%) patients with soft tissue tumours underwent wide excision (Table 1).

**Table 1:** Characteristics of patients operated during the study period at the Oxford Sarcoma Service (March 12 – May 12, 2020)

No. of patients	56 (23, bone tumours; 33, soft tissue tumours)
Median age (in years)	57 (18–87)
Gender (M/F)	30:26 (53.6:46.4%)
ASA grade	ASA I – 5 (8.9%) ASA II – 31 (55.3%) ASA III – 18 (32.1%) ASA IV – 2 (3.6%)
Details of surgeries	En bloc resection + replacement – 12 En bloc resection + reconstruction – 7 En bloc resection – 2 Metastatic fractures – 2 Soft tissue – wide excision – 33

*ASA* American Society of Anaesthesiologists; age is described as median

At the latest follow-up and assessment, 54 (96.4%) patients were recovering well post-surgery. Thirteen (23.2%) patients developed complications in the 30-day post-operative period.

## Complications

As per the Clavien-Dindo classification for complications, two (3.6%) patients had minor complications and 11 (19.6%) patients had major complications (Table 2). One patient developed superficial infection which resolved with regular dressings and one patient had postoperative pyrexia requiring extended hospital stay and this was managed symptomatically with an uneventful outcome.

**Table 2:** List of complications in patients (*n* = 56) following surgery

Overall	13 (23.2%)
Minor complications (CD – I and II)	
Superficial skin infection	1 (1.8%)
Post-operative pyrexia	1 (1.8%)
Major complications (CD – III, IV and V)	
Pulmonary embolism (PE)	4 (7.1%)
Wound re-explorations	4 (7.1%)
Escalation to intensive care due to ARDS secondary to COVID-19	3 (5.4%)
Mortality	2 (3.6%)

*CD* Clavien-Dindo classification for complications

Two of the three patients who required escalation to intensive care due to COVID-19 died

Four (7.1%) patients developed symptomatic pulmonary embolism (PE) following surgery, which was diagnosed using CT angiography. All of them were managed with low-molecular-weight heparin to have an uneventful outcome. Four (7.1%) patients developed wound complications requiring surgical re-exploration under anaesthesia. At the latest follow-up, the wounds of all these patients had healed well. Four (7.1%) patients, who developed specific symptoms related to COVID-19, tested positive for COVID-19 post-surgery. Three of them needed escalation to intensive care due to pulmonary complications and required mechanical ventilation as they developed acute respiratory distress syndrome (ARDS) and two (3.6%) of these patients, both females, died as a result of pulmonary complications.

None of the patients in ASA I category developed complications. One patient in ASA II category developed a minor complication (superficial skin infection). Ten patients in ASA III and both patients in ASA IV category developed complications. ASA grade was significantly (*p* < 0.001) associated with the number of complications, with higher ASA grades having increased complications.

Out of the 25 (44.6%) patients > 60 years, ten (17.8%) developed major complications and two (3.6%) developed minor complications. Only one (1.8%) patient in the > 60-year group (*n* = 31) developed a major complication (wound re-exploration). Ten (10/27 = 37%) patients operated at the index hospital (NOC) developed post-operative complications compared with three (3/29 = 10.3%) patients who were operated on at the COVID-free facility (Table 3). Three (3/27 = 11.1%) patients operated upon at the index hospital developed specific symptoms and tested positive for COVID-19 and subsequently two of them died due to associated complications. However, only one (1/29 = 3.4%) patient operated at the COVID-free facility tested positive for COVID-19 post-surgery and he recovered from the disease after symptomatic medical management in an isolation ward. He did not require escalation to intensive care. Patients < 60 years had significantly less number (3.2%) of complications compared with patients > 60 age group (48%) (*p* < 0.001); patients operated in a COVID-free facility had considerably less (*p* = 0.027) complications to those operated in the index hospital.

**Table 3:** Comparison between surgical procedures and their outcomes done at the index hospital and the designated COVID-free facility

	No. of surgeries (*n* = 56)	No. of patients contracting COVID-19	No. of patient deaths due to COVID-19	Complications
Index hospital	27	3	2	10 PE, 3 Re-exploration, 3 Escalation to ICU, 3 Postoperative pyrexia, 1
Designated COVID-free facility	29	1	0	3 Superficial Infection, 1 PE, 1 Re-exploration, 1

*PE* pulmonary embolism, *ICU* intensive care unit

## Discussion

These early results following surgery on BST during the COVID-19 pandemic were promising with majority of the patients having a favourable recovery outcome at the immediate follow-up. However, we had a complication rate of 23.2% and a mortality rate of 3.6% due to COVID-19. Patients more than 60 years old and patients under ASA categories III and IV were associated with increased complications following surgery. Surgery done at the COVID-free facility had favourable outcomes compared with surgery done at the index hospital.

The concern that cancer patients will have increased complications and a poorer outcome due to contracting COVID-19 following surgery still remains. A retrospective study which was conducted on 1099 patients with laboratory-confirmed COVID-19 and who are hospitalised in China through January 2020 showed that the cumulative risk of ICU admission, need for mechanical ventilation and death was 20.6% in surgical patients, compared with 3.6% of the general COVID-19 population [6]. The COVIDSurg Collaborative reporting on 30-day mortality and pulmonary complications following surgery in COVID-19 patients across 235 hospitals in 24 countries reported that more than half (51.2%) of them develop pulmonary complications with these patients having a 30-day mortality of 38% [5]. Hence, surgery on patients, especially those with cancer, is associated with significant risks. After the initial frenzy in healthcare systems following the pandemic, guidelines were issued and surgeons around the globe had more clarity with regard to managing their cancer patients. A recent report from the Tata Memorial Hospital in India where 494 cancer surgeries, combining scientific and administration rationale, were done across five weeks during the pandemic in a COVID-19 hotspot showed a major complication rate of 5.6% and no deaths due to COVID-19 [13]. This is the largest series of cancer patients operated upon during the pandemic to be reported so far and their results are reassuring. In this series of cases, BST surgeries (*n* = 27) accounted for 5.5% of their study group and all cases reported favourable outcomes. Our series of 56 cases of only BST is the largest series in sarcoma to the best of our knowledge at the time of writing this article. Our results, which are promising, also allowed us to reflect and introspect on our practice of cancer care during this pandemic and also formulate further recommendations (Table 4).

**Table 4:** Recommendations following early experience of surgeries on bone and soft tissue sarcoma (BST) patients during COVID-19 pandemic

- Patients with potential for curative surgery and patients under ASA I and ASA II to be prioritised - The use of a COVID-free facility for surgery has beneficial effects - Preoperative COVID-19 testing of all patients is essential - Regular mandatory testing of healthcare professionals -Intentional postponement of surgery on patients > 60 years of age and under ASA III and ASA IV categories - Adequate preoperative counselling to patients regarding the risk of pulmonary complications and mortality if they contract COVID-19	

The increased morbidity and mortality associated with cancer patients who contract COVID-19 is one of the factors influencing surgeons to defer surgery. Four (7.1%) patients who were deemed fit for surgery tested positive for COVID-19 following developing specific symptoms. Three patients developed pulmonary complications and required escalation of care. In spite of escalated intensive care treatment, two patients died due to ARDS secondary to COVID-19. The multicentre retrospective analysis of 205 cancer patients who contracted COVID-19 from Hubei, China, showed that 40 (20%) of them died during hospital admission with those with haematological malignancies having poorer outcomes than those with solid tumours [6]. Our mortality rate of 3.6% involving 56 tumours of only BST is encouraging compared with other cancers during the time of the pandemic. The two deaths in our series also imply that decision-making based on guidelines during the time of an evolving pandemic is fraught with possible risks. Both cases needed urgent surgery due to their presentation and aggressiveness of disease and further underline the fact that surgery on cancer patients during the pandemic is associated with risks. In elderly patients with increased co-morbidities, intentional postponement of surgery could be considered especially in endemic areas, as highlighted following initial experience on dealing with cancer cases in China at the start of the pandemic [1] and other recent published guidelines [14, 15]. Both patients who died in our series were females, which was different in relation to the earlier reports of male sex being a risk factor for higher mortality following cancer [5, 6].

The benefit of performing surgeries on cancer patients in a COVID-free hospital has been shown in our study. This facility had been structured in such a way that all patients and doctors are tested pre-operatively for COVID-19 [10]. Two weeks prior to the planned surgery date, the patient is advised to self-isolate at their residence or a place of stay, and 72 hours prior to surgery, the patient is brought to hospital to get COVID-19 swab tests at a separate enclosure away from the main facility. This exercise serves as an essential step to mitigate disease transmission. Restivo et al. [16], in a letter to the editor after observing the initial crisis in Italy, suggested that several hospitals need to be identified and treated as ‘COVID-free’ to manage cancer patients as such arrangements could go a long way in decreasing the risk of contagion and also protecting healthcare professionals from contracting the disease. However, one of the patients who got operated at the COVID-free facility in our hospital contracted the disease. This emphasises the importance of testing healthcare professionals who could represent asymptomatic carriers. After the first week of April, all healthcare professionals were tested and were only allowed into the COVID-free facility if they tested negative. At the time of writing this article, nearly 21 ‘virus-free’ centres are being set up by the NHS to manage cancer patients across England after the initial positive reports of their effectiveness across several centres including Royal Marsden and Guy’s and St. Thomas’ hospitals in London. The effectiveness of surgery in these centres across all cancers need to be analysed and would be useful in formulating further guidelines for cancer surgeries and BST surgeries [17]. However, even the use of COVID-free facilities is not infallible to contracting the disease as our study has shown with one patient contracting the disease at the designated facility. Healthcare professionals dealing with patients are also asymptomatic potential carriers of the virus and regular testing of them is essential to mitigate disease transmission. Mandatory regular testing of those involved with patients could be considered to overcome this pitfall.

The COVID-19 pandemic is a rapidly evolving phenomenon with situation changing day by day across the globe, making it difficult to bring out robust guidelines to manage patients. International inconsistency in implementation of protocols has exacerbated the dilemma of patient selection for cancer surgery. Our early results show that performing surgery on patients with sarcoma at the peak of the pandemic is fraught with the risk of disease contraction in a significant proportion (7.1%) of patients and half (3.6%) of them succumbing to it. Essential postponing of surgery in cases with associated co-morbidities and increased ASA grades (III and IV) to a suitable time later would be a prudent decision in case we encounter a second wave of the pandemic. Weighing the risk/benefit ratio coupled with the aggressiveness of disease would be ideal in listing patients with sarcoma for surgery.

We respectfully agree that our study is not without limitations. The wide variation of diagnosis and anatomical regions involved in surgery in BST makes it difficult to reach blanket conclusions and interpret results for BST as a whole. These are only results from a single centre, and multi-centric data across similar centres would add better perspective to the results following surgery and we intend to study this in the future. Our results could serve as a guide to similar centres dealing with BST. Not all healthcare professionals dealing with patients were tested pre-operatively in the beginning and the possibility of asymptomatic carriers still remained. However, as time passed, the testing capacity increased and all healthcare professionals were tested and only those who tested negative were patient-facing. Lastly, the current situation with the pandemic is a dynamic and rapidly evolving one; only time will tell the exact relevance of our results with respect to BST.

## Conclusion

Our results show that though surgery on BST cancers during the pandemic is associated with promising outcomes following prompt and efficient restructuring of departmental services, patients are also at risk of contracting COVID-19 in hospitals and succumbing to it. The use of a COVID-free facility for patients in ASA I and II categories and patients < 60 years of age is associated with better outcomes. Intentional delay of surgery in elderly patients and patients with co-morbidities (ASA III and IV) can be considered if we encounter a subsequent spike of the disease.

## Abbreviations

BST: bone and soft tissue tumours
ASA: American Society of Anaesthesiologists
NHS: National Health Service
BOOS: British Orthopaedic Oncology Society
BSG: British Sarcoma Group
NICE: National Institute for Health and Clinical Excellence
